# Supplementary material for: Depletion of individual dietary amino acids induce distinct metabolic and chromatin states
Source: J Biol Chem. 2025 Dec 17;302(2):111074. doi: 10.1016/j.jbc.2025.111074 (PMC12816912; doi:10.1016/j.jbc.2025.111074)
Supplement: Figure S1 [file mmc1.zip › jbc_111074_Haws2025_JBC_ExtendedDataFigure1_Final_mmc1.html]

 

 

 

 
 
 


 MOFA on dietary restriction 

 
 
 
 
 
 
 
 
 
 
 
 
 
 

 

 
 


 


 

 

 


 


 

 


 


 
 
 
 
 
 

 


 


 MOFA on dietary restriction 

 


 
 Data overview 
 Data consist of 60 mice from 4 datasets each consisting of 13929
transcripts, 15 physiological phenotypes, 103 metabolites, and 92
histone proteins. 
   
 
 
 Overview of the trained MOFA model 
 
 Correlation between Factors 
 The figure shows the correlation matrix between the latent Factors.
No correlation between Factors suggests a good model fit. 
   
 
 
 Variance decomposition 
 
 Variance decomposition by Factor 
 The plot shows the percentage of variance explained by the MOFA
Factors across different datasets. 
   
 
 
 Total variance explained per view 
 The plot shows the total percentage of variance explained across
different datasets. 
   
 
 
 
 
 Characterisation of Factors 
 
 Association analysis 
 The plot shows the Pearson correlation coefficient and its log10
adjusted p-values between the Factor values and the experimental
conditions. 
 
 Pearson correlation coefficient 
   
 
 
 log10 adjusted p-values 
   
 
 
 
 Factor values 
 The plot shows the violin plots of the Factor values colored by
experimental conditions per Factor. 
   
 
 
 Feature weights 
 The weights provide a score for each feature on each factor. Features
with no association with the corresponding factor are expected to have
values close to zero, whereas features with strong association with the
factor are expected to have large absolute values. The sign of the
weights indicates the direction of the effect: a positive weights
indicates that the feature has higher levels in the cells with positive
factor values, and vice-versa. 
 
 Ranked feature weights 
 The plots show the list of features ranked by their weights for each
Factor per datasets. 
 
 Factor 1 
 
 Metabolomics 
   
 
 
 RNAseq 
   
 
 
 HistoneProteomics 
   
 
 
 Physiology 
   
 
 
 
 Factor 2 
 
 Metabolomics 
   
 
 
 RNAseq 
   
 
 
 HistoneProteomics 
   
 
 
 Physiology 
   
 
 
 
 Factor 3 
 
 Metabolomics 
   
 
 
 RNAseq 
   
 
 
 HistoneProteomics 
   
 
 
 Physiology 
   
 
 
 
 Factor 4 
 
 Metabolomics 
   
 
 
 RNAseq 
   
 
 
 HistoneProteomics 
   
 
 
 Physiology 
   
 
 
 
 Factor 5 
 
 Metabolomics 
   
 
 
 RNAseq 
   
 
 
 HistoneProteomics 
   
 
 
 Physiology 
   
 
 
 
 Factor 6 
 
 Metabolomics 
   
 
 
 RNAseq 
   
 
 
 HistoneProteomics 
   
 
 
 Physiology 
   
 
 
 
 Factor 7 
 
 Metabolomics 
   
 
 
 RNAseq 
   
 
 
 HistoneProteomics 
   
 
 
 Physiology 
   
 
 
 
 Factor 8 
 
 Metabolomics 
   
 
 
 RNAseq 
   
 
 
 HistoneProteomics 
   
 
 
 Physiology 
   
 
 
 
 Factor 9 
 
 Metabolomics 
   
 
 
 RNAseq 
   
 
 
 HistoneProteomics 
   
 
 
 Physiology 
   
 
 
 
 Factor 10 
 
 Metabolomics 
   
 
 
 RNAseq 
   
 
 
 HistoneProteomics 
   
 
 
 Physiology 
   
 
 
 
 Factor 11 
 
 Metabolomics 
   
 
 
 RNAseq 
   
 
 
 HistoneProteomics 
   
 
 
 Physiology 
   
 
 
 
 Factor 12 
 
 Metabolomics 
   
 
 
 RNAseq 
   
 
 
 HistoneProteomics 
   
 
 
 Physiology 
   
 
 
 
 Factor 13 
 
 Metabolomics 
   
 
 
 RNAseq 
   
 
 
 HistoneProteomics 
   
 
 
 Physiology 
   
 
 
 
 Factor 14 
 
 Metabolomics 
   
 
 
 RNAseq 
   
 
 
 HistoneProteomics 
   
 
 
 Physiology 
   
 
 
 
 Factor 15 
 
 Metabolomics 
   
 
 
 RNAseq 
   
 
 
 HistoneProteomics 
   
 
 
 Physiology 
   
 
 
 
 
 Heatmap 
 The plot shows the heatmap of the feature weights clustered by rows
and columns for each Factor per datasets. 
 
 Factor 1 
 
 Metabolomics 
   
 
 
 RNAseq 
   
 
 
 HistoneProteomics 
   
 
 
 Physiology 
   
 
 
 
 Factor 2 
 
 Metabolomics 
   
 
 
 RNAseq 
   
 
 
 HistoneProteomics 
   
 
 
 Physiology 
   
 
 
 
 Factor 3 
 
 Metabolomics 
   
 
 
 RNAseq 
   
 
 
 HistoneProteomics 
   
 
 
 Physiology 
   
 
 
 
 Factor 4 
 
 Metabolomics 
   
 
 
 RNAseq 
   
 
 
 HistoneProteomics 
   
 
 
 Physiology 
   
 
 
 
 Factor 5 
 
 Metabolomics 
   
 
 
 RNAseq 
   
 
 
 HistoneProteomics 
   
 
 
 Physiology 
   
 
 
 
 Factor 6 
 
 Metabolomics 
   
 
 
 RNAseq 
   
 
 
 HistoneProteomics 
   
 
 
 Physiology 
   
 
 
 
 Factor 7 
 
 Metabolomics 
   
 
 
 RNAseq 
   
 
 
 HistoneProteomics 
   
 
 
 Physiology 
   
 
 
 
 Factor 8 
 
 Metabolomics 
   
 
 
 RNAseq 
   
 
 
 HistoneProteomics 
   
 
 
 Physiology 
   
 
 
 
 Factor 9 
 
 Metabolomics 
   
 
 
 RNAseq 
   
 
 
 HistoneProteomics 
   
 
 
 Physiology 
   
 
 
 
 Factor 10 
 
 Metabolomics 
   
 
 
 RNAseq 
   
 
 
 HistoneProteomics 
   
 
 
 Physiology 
   
 
 
 
 Factor 11 
 
 Metabolomics 
   
 
 
 RNAseq 
   
 
 
 HistoneProteomics 
   
 
 
 Physiology 
   
 
 
 
 Factor 12 
 
 Metabolomics 
   
 
 
 RNAseq 
   
 
 
 HistoneProteomics 
   
 
 
 Physiology 
   
 
 
 
 Factor 13 
 
 Metabolomics 
   
 
 
 RNAseq 
   
 
 
 HistoneProteomics 
   
 
 
 Physiology 
   
 
 
 
 Factor 14 
 
 Metabolomics 
   
 
 
 RNAseq 
   
 
 
 HistoneProteomics 
   
 
 
 Physiology 
   
 
 
 
 Factor 15 
 
 Metabolomics 
   
 
 
 RNAseq 
   
 
 
 HistoneProteomics 
   
 
 
 Physiology 
   
 
 
 
 
 
 
 Principal Component Gene Set Enrichment (PCGSE) 
 We used MSigDB_v6.0_C5_mouse gene sets for our enrichment analysis
consisting of 4436 biological pathways and 18360 genes. 
 
 Enrichment heatmap 
 The plots show the number of significant pathways per Factor for
upregulated gene sets (positive weight genes) and downregulated gene
sets (negative weight genes). 
 
 Enrichement for positive weights 
   
 
 
 Enrichement for negative weights 
   
 
 
 
 Enrichment results 
 The plots show top 50 upregulated gene sets and 50 downregulated gene
sets per factor. 
 
 Factor 1 
 
 Enrichement for positive weights 
   
 
 
 Enrichement for negative weights 
   
 
 
 
 Factor 2 
 
 Enrichement for positive weights 
   
 
 
 Enrichement for negative weights 
   
 
 
 
 Factor 3 
 
 Enrichement for positive weights 
   
 
 
 Enrichement for negative weights 
   
 
 
 
 Factor 4 
 
 Enrichement for positive weights 
   
 
 
 Enrichement for negative weights 
   
 
 
 
 Factor 5 
 
 Enrichement for positive weights 
   
 
 
 Enrichement for negative weights 
   
 
 
 
 Factor 6 
 
 Enrichement for positive weights 
   
 
 
 Enrichement for negative weights 
   
 
 
 
 Factor 7 
 
 Enrichement for positive weights 
   
 
 
 Enrichement for negative weights 
   
 
 
 
 Factor 8 
 
 Enrichement for positive weights 
   
 
 
 Enrichement for negative weights 
   
 
 
 
 Factor 9 
 
 Enrichement for positive weights 
   
 
 
 Enrichement for negative weights 
   
 
 
 
 Factor 10 
 
 Enrichement for positive weights 
   
 
 
 Enrichement for negative weights 
   
 
 
 
 Factor 11 
 
 Enrichement for positive weights 
   
 
 
 Enrichement for negative weights 
   
 
 
 
 Factor 12 
 
 Enrichement for positive weights 
   
 
 
 Enrichement for negative weights 
   
 
 
 
 Factor 13 
 
 Enrichement for positive weights 
   
 
 
 Enrichement for negative weights 
   
 
 
 
 Factor 14 
 
 Enrichement for positive weights 
   
 
 
 Enrichement for negative weights 
   
 
 
 
 Factor 15 
 
 Enrichement for positive weights 
   
 
 
 Enrichement for negative weights 
   
 
 
 
 


 
 

 

 

 

 

 

 

 
 

 
 
