# Supplementary material for: Depletion of individual dietary amino acids induce distinct metabolic and chromatin states
Source: J Biol Chem. 2025 Dec 17;302(2):111074. doi: 10.1016/j.jbc.2025.111074 (PMC12816912; doi:10.1016/j.jbc.2025.111074)
Supplement: Supplementary Material 1 [file mmc10.pdf]

Supplemental Figure 1 - Differential p62 and LC3B expression following dietary EAA depletions.

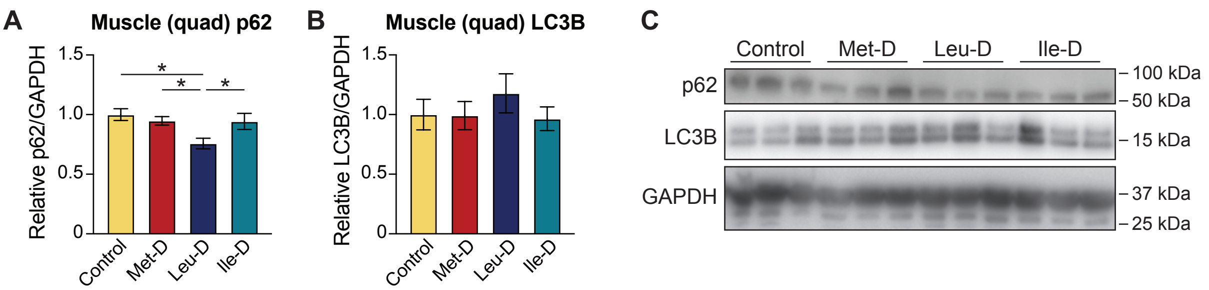

Supplemental Figure 1: Differential p62 and LC3B expression following dietary EAA depletions. (*A-B*) Bar graphs depicting muscle (quadricep) p62 and LC3B protein levels normalized to GAPDH. (*C*) Representative western blot images of those used for the quantification depicted in panesl A and B. Error bars = SEM; N = 12; * = p-value < 0.05 as measured via Student's t-test.

Supplemental Figure 2 - Select Ile-D specific gene expression changes following dietary EAA depletions and rapamycin treatment.

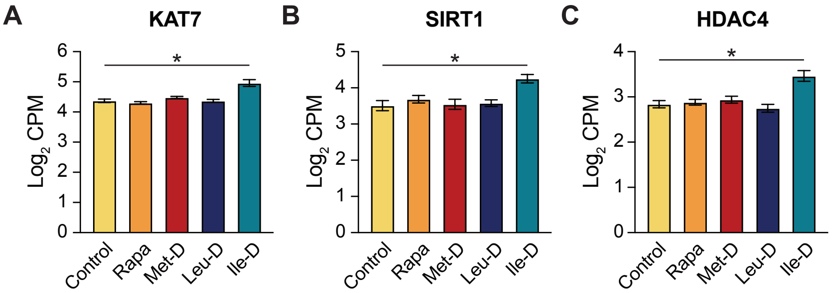

Supplemental Figure 2: Select Ile-D specific gene expression changes following dietary EAA depletions and rapamycin treatment. (*A*-*C*) Bar graph depicting $\log_2$ CPM values calculated from bulk liver RNA-sequencing data. Error bars = SEM; N = 12; * = adj. p-value < 0.05 as measured via *edgeR* and *limma*.
