# Supplementary material for: Depletion of individual dietary amino acids induce distinct metabolic and chromatin states
Source: J Biol Chem. 2025 Dec 17;302(2):111074. doi: 10.1016/j.jbc.2025.111074 (PMC12816912; doi:10.1016/j.jbc.2025.111074)
Supplement: Supplementary Material 2 [file mmc11.docx]

**Supplementary Table Legends**

**Table S1:** *M. musculus* diet compositions.

**Table S2:** Liver bulk RNA-sequencing results summary.

**Table S3:** GO-BP gene enrichment for shared differentially expressed genes across all EAA-depletion conditions.

**Table S4:** GSEA pre-ranked results using genes which are differentially expressed in response to a single EAA-depleted condition.

**Table S5:** Liver metabolomics data summary.

**Table S6:** Liver histone proteomics stoichiometry data summary.

**Table S7:** HepG2 metabolomics data summary.

**Table S8:** HepG2 histone proteomics stoichiometry data summary.
